# Lipoma in the hypopharynx: a case report

**DOI:** 10.1016/j.bjorl.2026.101764

**Published:** 2026-01-29

**Authors:** Diego Guzman Rodrigues, Rynat Dasaev Oliveira Chagas, Natália Matos Muricy, Raissa Damasceno Barreto da Silva

**Affiliations:** a Hospital Santo Antônio, Department of Otorhinolaryngology, Salvador, BA, Brazil; b Universidade de São Paulo, São Paulo, SP, Brazil

## Introduction

Lipomas are the most common benign tumors of mesenchymal origin in the body. Only 15% of them, however, are in the head and neck region and when present they are usually in the subcutaneous region.^1^ Lipomas in deeper locations, such as the hypopharynx, are uncommon, with few cases reported in the literature.^2^ Symptoms vary according to the size of the tumor, possible manifestations include dysphagia, pharyngeal globus, dysphonia, cough, dyspnea and snoring.^3^ The aim of this article is to present a rare case of hypopharyngeal lipoma and analyze the main characteristics that help in its diagnosis.

## Case report

Patient B.F.N, 66-years-old, male, complaining of dysphonia, dysphagia when eating solid food and dyspnea when lying down for approximately one year after admission. He denied previous clinical comorbidities or smoking history.

**Fig. 1:**
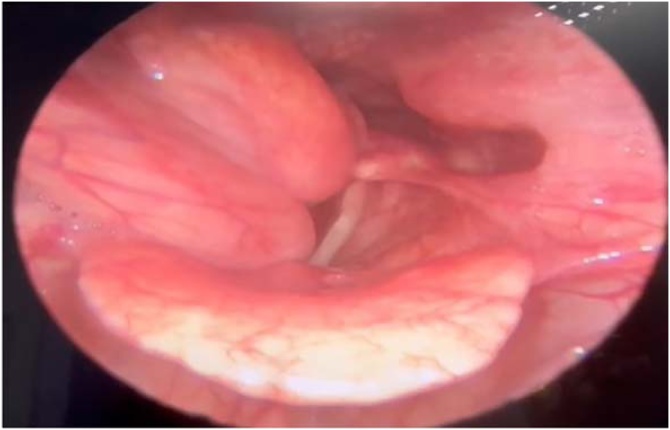
Videolaryngoscopy showing a lesion on the right.

Videolaryngoscopy revealed the presence of a smooth, bilobed mass protruding from the right posterolateral wall of the hypopharyngeal region to the right, partially obstructing the glottic lumen during inspiration ‒ other structures of the larynx and pharynx showed no other changes (Fig. 1).

**Fig. 2:**
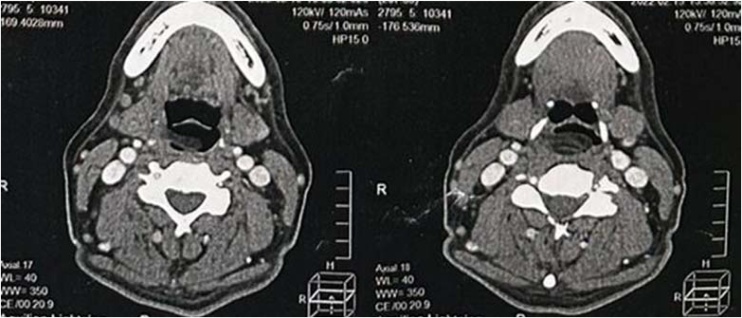
Neck tomography with contrast showing a lesion.

The tomography of the neck showed an expansive formation with a density of adipose tissue without enhancement after the use of contrast, extending from the right paraglottic space and posterior wall towards the laryngeal lumen with the reduction of it. The tumor had a size of approximately 3.0 × 2.0 × 1.2 cm, with appearance compatible with lipoma (Fig. 2). No enlargement of cervical lymph nodes was observed.

The patient was then referred for surgical excision. Suspension laryngoscopy was performed with exposure of two lobulated lesions with a smooth surface and pedunculated with the lateral wall of the hypopharynx on the right, adjacent to the epiglottis. Complete excision of them was performed with the aid of monopolar electrocautery. The procedure went without complications, and the patient was extubated without difficulty. He remained hospitalized for one day, being discharged with good acceptance of an oral diet and comfortable without the use of oxygen therapy.

Histopathological examination using hematoxylin-eosin staining revealed mature adipose tissue arranged in lobules separated by thin fibrous septa, without evidence of cellular atypia or mitotic figures, consistent with a diagnosis of benign lipoma. Although the histopathological image could not be included due to institutional limitations, the microscopic findings were typical for lipoma.

**Fig. 3:**
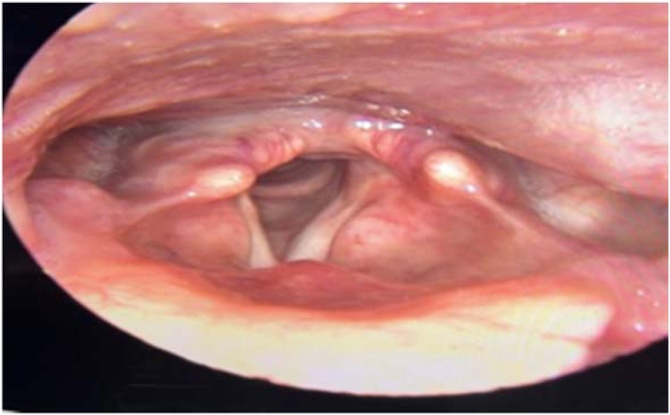
Post-operative endoscopic appearance.

The patient presented good postoperative evolution, with complete improvement of the symptoms and videolaryngoscopy performed two months after the procedure showed complete tissue healing (Fig. 3).

## Discussion

Lipomas are benign tumors of mesenchymal origin that grows slowly and normally have no life-threatening repercussions. They are more common in areas with a high fat content and account for up to 5% of benign tumors throughout the body and 15% of neck tumors. In the respiratory tract, however, they are present in less than 1% of cases and can even lead to obstructive symptoms. The most affected sex is male in a ratio of 3.6:1; with a more frequent appearance in the 40-year-old age group.

Hypopharyngeal lipomas are extremely rare, with fewer than 100 cases reported in the literature to date. They represent less than 0.6% of all benign tumors in the upper aerodigestive tract, and the hypopharynx is among the least common sites.^4^

The limited number of documented cases amplifies the clinical relevance of reporting new occurrences, as they contribute valuable information regarding presentation, diagnosis, and surgical management. Moreover, due to their insidious growth and submucosal development, these tumors may go unrecognized until they cause significant airway or swallowing symptoms. For this reason, despite its rarity, hypopharyngeal lipoma should always be considered in the differential diagnosis of submucosal masses in the upper airway.

There are four differential diagnoses to consider when it comes to tumors with fat density in the hypopharynx: Lipoma, liposarcoma, lipoblastoma and hibernoma. It is crucial to distinguish between these injuries to provide appropriate monitoring and intervention for patients.^5^

The initial approach for patients with obstructive upper airway complaints should be to perform a videolaryngoscopy. If expansive tumors are seen in this region, the exam of choice for diagnostic complementation is a computed tomography of the neck with contrast. It can be also complemented with magnetic resonance imaging, when available. However, a definitive diagnosis can only be made by anatomopathological analysis.

After excluding malignancy of the tumor, it is recommended to avoid radical surgical approaches that could result in greater morbidity and impair the patient's quality of life.^2^ It is concluded that the oral access via direct laryngoscopy is a good choice for the procedure. Ideally, should be excised most of the lesion without compromising cervical structures and the material should be sent for histological analysis.

This initial measure promotes a considerable improvement in the patient's obstructive symptoms, as well as a good return of laryngopharyngeal functions. Appropriate surgical treatment for these injuries has a low recurrence rate.^4^

## Conclusion

Lipoma is a differential diagnosis of hypopharyngeal tumors, such as the case report presented in this article. Although rare, it should be considered as a hypothesis in the case of tumors with fat density on complementary imaging tests. Surgical treatment is recommended, with resections that preserve the structures and functionality of the laryngopharynx, with a high success rate.

## ORCID ID

Diego Guzman Rodrigues: 0000-0002-3615-957X

Rynat Dasaev Oliveira Chagas: 0000-0002-4509-0623

Natália Matos Muricy: 0009-0004-4703-7553

Raissa Damasceno Barreto da Silva: 0009-0008-8806-9331

## Funding

The authors received no specific funding for this work.

## Conflicts of interest

The authors declare no conflicts of interest.

## Data availability statement

The authors declare that all data are available in repository.
